# The power of multimodal antidepressants: decrease of soluble ST2 blood level in patients with depression and comorbid heart failure

**DOI:** 10.1192/j.eurpsy.2023.1097

**Published:** 2023-07-19

**Authors:** A. K. Sikora, S. Fedorov, M. Vynnyk, M. Pustovoyt

**Affiliations:** 1 Psychiatry, addiction and medical psychology; 2 Therapy and family medicine- post graduate, Ivano-Frankivsk National Medical University, Ivano-Frankivsk, Ukraine

## Abstract

**Introduction:**

Nowadays the disorders of Mental sphere are still the clinically significant disturbances in an individual’s cognition, emotional regulation, and behaviour. They are usually associated with distress or impairment in important areas of functioning. In recent years, more than 280 million people were living with depression, including 23 million children and adolescents. Depression is different from usual mood fluctuations and short-lived emotional responses to challenges in everyday life. Heart failure (congestive heart failure) is the leading cause of hospitalization in people older than 65. Soluble ST2 is regarded as a key molecule regulating immune system as well as cell proliferation. Elevated serum concentrations of soluble ST2 have been reported in patients with neuropsychiatric disorders, suggesting pathophysiological roles of soluble ST2 in behavioral phenotypes.

**Objectives:**

measurement of the amount of ST2 molecule levels in the blood of patients with depressive disorders and comorbid heart failure.

**Methods:**

there were examined 180 patients with depression of varying severity together with the syndrome of HF of ischemic genesis, FC II-IV (NYHA). Depressive disorders were diagnosed and determined using the Hamilton Depression Rating Scale (HDRS) with the result in 20 and more points. Transthoracic echocardiography was performed; the plasma content of soluble ST2 (sST2) and the titers of the N-terminal fragment of the brain natriuretic peptide (NT-proBNP) in the blood were determined by ELISA. Statistical analysis was performed using the standard software package “Statistica for Windows 12.0” (StatSoft, Tulsa, OK, USA).

**Results:**

The average age of the examined patients was (69.33 ± 10.44) years; among them - 64 women (35.5%). It was found that the levels of soluble ST2 and NT-pro-BNP in the blood of patients with depressive disorders and HF decreased with prescribing of 3 months therapy of multimodal antidepressant. After the treatment using vortioxetine, the majority of patients with HF and depressive disorders of moderate intensity - 111 (61.6%) people –noted a reduction in symptoms of both depression and heart failure. Univariate correlation analysis showed a direct relationship between soluble ST2 levels in the blood of the examined patients and the reduction of depressive symptoms (r^s^= 0.33; p = 0.041); blood content of NT-pro-BNP (r^s^ = 0.51; p = 0.015); resting heart rate (r^s^ = 0.31; p = 0.011) and feedback from LV EF (r^s^ = -0.39; p = 0.043).

**Conclusions:**

Patients with depressive disorders and concomitant heart failure that are treated with multimodal antidepressants (vortioxetine) are more likely to have decreased levels of soluble ST2 molecule in the blood, that explain the anti-inflammatory together with anti depressive effect of this medication.

**Disclosure of Interest:**

None Declared

